# Metric System

**Published:** 1878-09

**Authors:** 


To convert grains or minims into centigrammes, multiply them by 6; to convert drachms into grammes, multiply them by 4; to convert ounces into grammes, multiply them by 32. For instance:

| | | | |
|---|---|---|---|
| ℞ Quiniæ Sulph | grs. xxx | 1 | 80 |
| Acidi Sulphurici dil | ʒ iss | 6 | 00 |
| Ferri Sulphatis | ʒ ss | 2 | 00 |
| Mix. Aq. Fort | ℥ iij | 96 | 00 |

It will be well for our readers to familiarize themselves with this, for the time is coming, and even now is, where ignorance thereof will be culpable.—*Mich. Med. News.*

